# Assessing the Oxidative Degradation of N-Methylpyrrolidone (NMP) in Microelectronic Fabrication Processes by Using a Multiplatform Analytical Approach

**DOI:** 10.1155/2020/8265054

**Published:** 2020-03-04

**Authors:** Gavin Lennon, Shannon Willox, Ragini Ramdas, Scott J. Funston, Matthew Klun, Robert Pieh, Stewart Fairlie, Sara Dobbin, Diego F. Cobice

**Affiliations:** ^1^Queen's University Belfast, School of Chemistry and Chemical Engineering, David Keir Building, Stranmillis Road, Belfast, Antrim BT7 1NN, UK; ^2^Seagate Technology PLC, Springtown Industrial Estate, Londonderry BT48 0LY, UK; ^3^Seagate Technology PLC, Bloomington, MN 55435, USA; ^4^Ulster University, Centre of Molecular Biosciences, Biomedical Science Research Institute, Mass Spectrometry Centre, Cromore Road, Coleraine BT52 1SA, UK

## Abstract

During the construction of recording head devices, corrosion of metal features and subsequent deposition of corrosion by-products have been observed. Previous studies have determined that the use of N-methylpyrrolidone (NMP) may be a contributing factor. In this study, we report the use of a novel multiplatform analytical approach comprising of pH, liquid chromatography/UV detection (LC/UV), inductively coupled plasma optical emission spectroscopy (ICP-OES), and LC/mass spectrometry (LC/MS) to demonstrate that reaction conditions mimicking those of general photoresist removal processes can invoke the oxidation of NMP during the photolithography lift-off process. For the first time, we have confirmed that the oxidation of NMP lowers the pH, facilitating the dissolution of transition metals deposited on wafer substrates during post-mask and pre-lift-off processes in microelectronic fabrication. This negatively impacts upon the performance of the microelectronic device. Furthermore, it was shown that, by performing the process in an inert atmosphere, the oxidation of NMP was suppressed and the pH was stabilized, suggesting an affordable modification of the photolithography lift-off stage to enhance the quality of recording heads. This novel study has provided key data that may have a significant impact on current and future fabrication process design, optimization, and control. Results here suggest the inclusion of pH as a key process input variable (KPIV) during the design of new photoresist removal processes.

## 1. Introduction

**Figure 1:**
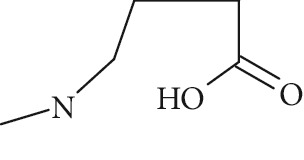
Semiconductor device fabrication process used to create integrated circuits that are present in electrical and electronic devices. Adapted from Dean et al. [2].

Semiconductor device fabrication is the process used to create integrated circuits that are present in electrical and electronic devices [1]. The fabrication process shown in Figure 1 begins with a wafer of semiconductor material and includes a sequence of photographic and chemical processing steps during which electronic circuits are gradually created on the wafer substrate [2]. Advanced semiconductors may contain billions of transistors on a layer of silicon the size of a square centimeter, so manufacturing must be rigorously controlled and conducted with great precision to achieve features at the nanoscale [3].

The production of semiconductors is reliant on the use of photolithography processes [4] which utilize chemical formulations in specialized manufacturing tools that pattern integrated circuits with linewidths that may be only ten nanometres or less in width.

Lithography accounts for over one-third of the total production costs in the fabrication of microelectronics [5] as this stage is prone to generating defects [6]. However, defects arising from chemical phenomena occurring in the photoresist and photoresist stripper chemicals are less studied.

Photoresist formulations are typically comprised of five or more individual chemical components, including a polymeric resin, radiation sensitive compound, and a solvent [6] that must work together in concert to receive a light image and delineate the desired integrated circuit pattern on the wafer surface [2]. The photoresist strip step must be accomplished in a manner that completely and uniformly removes the residual photoresist, without adversely impacting the surfaces of the materials comprising the underlying wafer substrate [7, 8]. One method to remove the photoresist and Bottom Anti-Reflective Coatings (BARC) involves the use of the liquid N-methylpyrrolidone (NMP) as a solvent. NMP is only slowly oxidized by air, and it is very hygroscopic [9]. However, NMP has been shown to oxidize in the presence of transition metals under nontropospheric conditions by well-known catalytic mechanisms [9–12], as well as in the presence of UV-light and hydroxyl radical initiators [13].

During previous studies by Seagate Technology's photolithography engineers, issues relating to fluctuations in the resistance of magneto-resistive recording heads (MRR) were observed (Supplementary [Supplementary-material supplementary-material-1]) to result in the failure of the drive to read the servo patterns of hard disk media. Analysis of the photolithography engineering process indicated that the issue was the result of transition metal feature corrosion at the contact reader stack occurring during the photoresist lift-off process (unpublished data). In this instance, the chemical in question was NMP which is known to dissolve certain transition metals [13]. There are various hypotheses regarding potential mechanisms of action of this chemical, which ultimately lead to the destruction of crucial recording head components.

Reist and George [14] provided valuable insights into the dissolution mechanism of copper under aqueous conditions. In their study, they found that the presence of molecular oxygen (O_2_) enabled the formation of a CuO surface layer which protects the underlying copper metal. However, in the presence of a protic solution, the H^+^ ions have a high affinity for the oxygen component of the copper(II) oxide surface layer, and so two equivalents of protons readily combined with the oxygen of the surface oxide to generate water and solvated Cu(II^+^) ions. This exposes the underlying copper metal allowing further oxidation-dissolution process to proceed. Based on this and other work [15] relating to the impact of system pH on metal dissolution, we hypothesize that although NMP itself has a considerably basic pH, exposure of the chemical to work-in-progress (WIP) wafers may initiate a chemical transformation which acts to solvate transition metals on the surface of the wafer, whilst simultaneously lowering the pH of the system—thus accelerating the dissolution process and/or the NMP degradation process.

Here, we report the development and application of a novel multiplatform analytical approach which combines the use of pH, LC/UV, ICP-OES, and LC/MS methods to assess NMP oxidative degradation pathways and monitor its progression within photolithography lift-off processes.

## 2. Materials and Methods

### 2.1. Chemicals

N-Methylpyrrolidone (HPLC grade, ≥99%), N-methylsuccinimide (99%), 1-(2-hydroxymethyl)-2-pyrrolidone (98%), 2-pyrrolidone (≥99%), succinimide (99.1%), N-hydroxymethylpyrrolidone (R&D synthesized—no purity data), cobalt(II) chloride hexahydrate (98%), and formic acid (98% v/v) were all obtained through Sigma Aldrich, Haverhill, UK. Silver nitrate aqueous solution (0.02 M, ≥99%) was obtained through VWR Chemicals, Lutterworth, UK. Sodium Y-52 zeolite was obtained through Honeywell Fluka, Cambridge, UK. Oxygen (99.999%), nitrogen (99.999%), acetonitrile (HPLC grade, ≥99%), water MiliQ (18 Ω conductivity), methanol (HPLC grade, ≥99%), buffer solution pH 10 (pH 10 ± 0.01 at 25°C, 0.1 M), and buffer solution pH 7 (pH 7 ± 0.04 at 25°C, 0.1 M) were obtained through Schlötter, Co. Kildare, Ireland. Deuterated water: Cambridge Laboratories, INC. Lot #6K-328, Cambridge, UK. ICP-OES elemental standard solutions were obtained through Thermo Scientific, Altrincham, UK.

### 2.2. pH Analysis

pH analysis was conducted using an EasyClean Solvotrode electrode (Metrohm UK) with a LiCl in ethanol (1 M) reference electrolyte and a 716 DMS Titrino unit (Metrohm UK). pH data were analyzed using Tiamo 2.4 (Metrohm UK).

### 2.3. Inductively Coupled Plasma Optical Emission Spectroscopy (ICP-OES) Analysis

ICP-OES analysis was conducted using an iCAP 7400 ICP-OES instrument (Thermo Fisher Scientific). NMP samples were dissolved in deionized water prior to analysis (10% v/v). Plasma conditions were as follows: RF power: 1250 W, auxiliary gas flow: 0.5 L/min, coolant gas flow: 12 L/min, nebulizer gas flow: 0.5 L/min, and nebulizer gas pressure: 270 kPa. Spectra were analyzed using Qtegra ISDS (Thermo Fisher Scientific).

### 2.4. Inductively Coupled Plasma Optical Emission Spectroscopy (ICP-OES) Calibration

The iCAP 7400 ICP-OES instrument was calibrated to the following elements: aluminum, arsenic, cobalt, chromium, copper, iron, gallium, nickle, platinum, and silicon. A high concentration working standard (10 mg/L) was prepared by dissolving the respective elemental standard (1 mL) in concentrated HNO_3_ (2 mL), the acidic solutions were combined and the resulting solution was diluted to 100 mL with deionized water. A low concentration working standard (0.1 mg/L) was prepared by dissolving an aliquot of the high concentration working standard (1 mL) in concentrated HNO_3_ (2 mL), the resulting solution was then diluted to 100 mL with deionized water. The instrument was then calibrated using NMP-based standard solutions ([Supplementary-material supplementary-material-1]).

### 2.5. Cobalt-Sodium Y-Zeolite Preparation

The zeolite-supported cobalt material was prepared as outlined previously [10]. Cobalt(II) chloride hexahydrate (2 × 10^−2^ mol) was added to a 500 mL conical flask containing deionized water (200 mL) and equipped with a magnetic stir bar. Sodium Y-zeolite (10 g) was added to the reaction flask and the reaction mixture was allowed to stir at 70°C for 24 h. The resulting pink solid was filtered under vacuum and washed with deionized water until no chloride was detected upon addition of silver nitrate solution (0.1 N). The solid was then dried in vacuo for 48 h at 150°C in a vacuum oven to give a pale purple/blue solid (8.8329 g). The dried solid was then transferred to a vacuum desiccator and allowed to cool to room temperature in vacuo after which it was ready for use.

### 2.6. Catalytic Oxidation of NMP in Oxygen

The procedure was based on previous work [16]. To a three-neck round bottom flask (RBF) equipped with a magnetic stir bar as shown in ([Supplementary-material supplementary-material-1]), N-methylpyrrolidone was added (50 mL). A three-way separator attachment, with an oxygen-filled balloon fitted to one nozzle and a vacuum line fitted to the other, was inserted into the central opening of the flask while the other two openings were sealed with glass stoppers—all openings were sealed with vacuum grease and parafilm. Dry cobalt-sodium Y-52 zeolite (0.2629 g) was added to the reaction flask to yield a light blue heterogeneous mixture. The reaction vessel was evacuated and flushed with oxygen (99.999%) three times before being placed in a water bath with a temperature maintained between 75°C and 80°C and allowed to stir for approximately 164 h. Multiple color changes were noted throughout the reaction and recorded in order as light blue, murky green, light brown, and dark brown/black. No solid catalyst was recovered at the end of the reaction. Daily samples of the reaction (1 mL) were extracted and pipetted into a 250 mL beaker containing deionized water (99 mL) and stirred. An aliquot of the resulting aqueous solution (1.5 mL) was submitted for LC-UV and LC/MS analysis. After the samples were drawn, the system was evacuated and flushed with oxygen gas three times.

### 2.7. Catalytic Oxidation of NMP under Nitrogen Atmosphere (Control)

Reaction was conducted using same conditions as previously described in Section 2.6 except that one nozzle of the three-neck RBF was filled with nitrogen (99.999%) instead of oxygen.

### 2.8. Liquid Chromatography/Ultraviolet Detection (LC/UV) Analysis

Standards were prepared as follows: 1 mL of standard solution (1 M) was transferred into a 100 mL volumetric flask, 90 ml of water was added, vortex mixed for 5 sec and made up to volume with water to achieve a 0.01 M solution. LC-UV analysis was conducted using a Thermo Fisher Scientific Dionex Ultimate 3000 RSLC (Thermo Fisher Scientific, US) in gradient mode. The column temperature was set at 40°C, and the UV detector was set at 230 nm. Mobile phases were as follows: A: water, B: acetonitrile with the injection volume of 10 *μ*L, and the LC system was operated in gradient mode ([Supplementary-material supplementary-material-1]). Thermo Scientific Acclaim RSLC 120 C18 2.2 *μ*m 120 Å 3.0 × 100 mm (Thermo Fisher Scientific, US) was used as the LC column. All LC-UV data were processed using Thermo Scientific Chromeleon 7.2 CDS software (Thermo Fisher Scientific, US).

### 2.9. Liquid Chromatography/Mass Spectrometry (LC/MS) Analysis

Liquid chromatography/mass spectrometry analysis was conducted using a Micro-LC Dionex RLSC nano Ultimate 3000 adapted with a micro flow meter coupled with a LTQ-XL-Orbitrap XL (Thermo Fisher Scientific) mass spectrometer. Same gradient was used as per [Supplementary-material supplementary-material-1] except for the addition of an acidic modifier (formic acid 0.01% (v/v)) to both mobile phases and micro LC Column Acclaim RSLC 120 C18 2.2 *μ*m 120 Å1.0 × 50 mm (Thermo Fisher Scientific) was used at a constant flow of 45 *μ*L/min. Ionization was electrospray (ESI) in positive ion mode with a mass range of 50–200 Da. ESI conditions were as follows: ion spray voltage: 4.6 (V), capillary temperature: 280°C, sheath gas flow: 20 (Arb) auxiliary gas flow: 8 (Arb), collision energy (for collision induced dissociation (CID) MSn) ramp mode from 15–50 (V), and mass resolution mode IT-FT: 30,000 resolution power (RP). Mass spectra were processed using Xcalibur version 2.2 (Thermo Fisher Scientific).

### 2.10. Deuterium Exchange Analysis

Deuterium exchange analysis was performed using the same LC platform as per Section 2.8. D_2_O +0.01% formic acid (v/v) was used as mobile phase A.

## 3. Results and Discussion

### 3.1. Assessment of NMP pH Variation under Standard Operating Conditions

**Figure 2:**
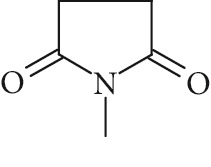
(a) Variation of the pH of NMP with time and upon use in the photolithography lift-off process under standard operating conditions (aerial atmosphere) using a Veeco PSP M3303 Trilennium solvent processor. (b) Concentration of various elements within the WIP wafer build in NMP over the course of its lifetime in the photolithography lift-off process under standard operating conditions + N_2_ blanket. WIP rate of 5000 wafer per month. (c) pH of NMP over its lifetime in the photolithography lift-off process under standard operating conditions + N_2_ blanket. WIP rate of 5000 wafer per month. (d) Variation of NMP pH under oxygen-rich and nitrogen-rich conditions in the presence of Co-Na Y-52 zeolite at 80°C.

The stability of the pH of NMP used in the photolithography lift-off process was first observed over a period of time under standard operating conditions in which the chemical is exposed to air. The pH remained nearly constant, ranging between pH 10.39 and 9.69 over a period of 144 h. After 168 h, the NMP was exposed to 328 work-in-progress (WIP) wafers over a period of 48 hours at 80°C, which resulted in a rapid shift in pH from 9.69 to 6.82 (Figure 2(a)). Given the observed stability of the pH of NMP at the standard operating temperature of 80°C, it can be concluded that the shift in pH was the result of an unknown interaction between NMP and on-wafer materials such as unreacted photoresist or transition metals from the constructed electromagnetic features.

### 3.2. ICP-OES Analysis of NMP under Inert Operating Conditions

The dissolution of elements from the surface of WIP wafers during the photolithography lift-off process was verified by using ICP-OES analysis to observe the variation in elemental content within the NMP over its lifetime in the fabrication process (Figure 2(b)).

Over a period of approximately three months, a steady increase in the concentration of cobalt dissolved in the NMP was observed whilst the concentration of the remaining elements investigated saw only relatively small increases over the same period. The pH of the NMP during this experiment showed an immediate rapid decrease from pH 11.28 to 7.10 within a period of 144 h in the fabrication process followed by a slower rate of decline to pH 5.65 after a period of 552 h. Proceeding this initial period of rapid pH decline, the system became more stable with a relatively low rate of pH decline over the remainder of the NMP's lifetime in the fabrication process with a minima of pH 4.83 observed after 2328 h. Based on these results, there is a clear correlation between the affinity for cobalt dissolution and the pH of NMP. High cobalt levels were observed in the solvent after exposure to 15,000 WIP wafers whilst the pH of the NMP was shown to rapidly decrease in a relatively short initiation period. This pH decline in correlation with the increased transition metal concentration dissolved within the NMP over time is in agreement with the consensus of current literature that an acidic media facilitates the dissolution of transition metals [17, 18]. Furthermore, although the rate of cobalt dissolution appears to dominate in comparison to the other metals monitored by ICP-OES analysis, there are currently no insights into the role of a purely organic medium in the dissolution mechanism of such metals; thus, the reason for preferential solvation of cobalt is unknown. However, the kinetics and mechanism of cobalt dissolution have been studied extensively in acidic aqueous media and in the presence of organic additives [19–21]. Under such aqueous acidic conditions, the dissolution of cobalt and other transition metals proceeds via the interaction of metal oxide sites with H^+^ ions and/or H^−^ anion pairs with organic additives accelerating the process via complexing with the metal sites. This combined study using ICP-OES analysis and pH measurement has provided crucial insights into the source of acidification in the system. Given that a nitrogen blanket was used in these ICP-OES/pH trials, effects of aerial exposure such as excessive water and CO_2_ content [14] can be discounted; hence, the acidification of the chemical environment was purely a phenomenon of the NMP-wafer interaction.

### 3.3. pH Analysis of NMP Degradation in the Presence of Cobalt

With the considerable amount of cobalt dissolved by NMP during the ICP-OES experiments, small scale monitoring was then used to observe the role of cobalt in the decline of the pH of NMP. To avoid the effects of other WIP wafer materials, cobalt was suspended on a standard ion-exchange type Y zeolite support and placed in NMP under standard photolithography lift-off operating conditions in the presence of both oxygen-rich and nitrogen-rich environments. Under oxygen-rich conditions, a period of rapid pH decline was observed over the initial 44 h with the systems pH dropping from 8.95 to 4.48. This was followed by a continued period of pH decline but at a much slower rate with a minima of pH 3.47 being observed after 164 h. Comparatively, under nitrogen-rich conditions this initial period of rapid pH decline was almost eliminated with pH dropping from 8.06 to 6.71 over the initial 44 h. Additionally, the pH of NMP under nitrogen-rich conditions remained stable in the range of pH 6.8 to 6.4 for the remainder of the reaction period (Figure 2(c)). Hence, it can be seen that in an oxygen-rich environment the pH of NMP rapidly declines to an acidic pH in a short period of time. In contrast, when the same system was saturated with nitrogen, the initial rapid pH decline of NMP was almost eliminated. Furthermore, the rate of pH decline was significantly suppressed by the pH stabilized-NMP in a weakly acidic pH range of 6.8 to 6.4. This was also observed in the pre-photolithography lift-off process ICP-OES experiments in which the pH of the NMP used in this fabrication step remained stable over a period of months, reaching a minimum of pH 4.83 after approximately three months and exposure to 15,000 WIP wafers. It was then concluded that oxygen plays a crucial role in the degradation of NMP leading to the observed acidic solution, thus prompting the use of LC/UV and LC/MS techniques to assess the acidification mechanism at the molecular level.

### 3.4. Catalytic Oxidation Assessment by LC/UV

**Figure 3:**
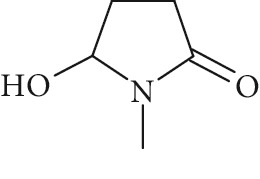
LC/UV chromatogram of NMP. (a) Standard; (b) after catalytic oxidation, NMP was mainly degraded into RRT 0.92 (10.5%), NMS (15%), and RRT 1.16 (11.4%) and other minor degradation products at RRT 0.27 (2.5%), RRT 1.52 (1.2%), and RRT 1.82 (0.8%). (c) Degradation was substantially suppressed under nitrogen atmosphere with RRT 0.92 (∼4.3%), and NMS (∼2.8%) being the main degradation products. NM4ABA: N-methyl-4-aminobutanoic, NMS: N-methylsuccinimide, 5-HNMP: 5-hydroxy-N-methylpyrrolidone, FP: 1-formyl-2-pyrrolidone, 2-AP: 1-(2-Hydroxyethyl)-2-pyrrolidone, 2-NEP: 1-acetyl-2-pyrrolidone, NEP: N-ethylpyrrolidone and NMP: N-methylpyrrolidone. (d–h) Known NMP degradation products and process impurities standard solution mix. NMP, NMS, 2P: 2-pyrrolidone, NHEP: N-hydroxyethyl-2-pyrrolidone, NHMP: N-hydroxy-N-methyl-2-pyrrolidone.

After exposure to the Co-Na Y-52 zeolite, NMP was mainly degraded into relative retention time (RRT) 0.92 (10.5%), NMS (15%), and RRT 1.16 (11.4%) and other minor degradation products at RRT 0.27 (2.5%), RRT 1.52 (2.1), and RRT 1.82 (1.5%) as shown in Figure 3(b). Degradation was substantially suppressed under nitrogen atmosphere with RRT 0.92 (∼4.3%) and NMS (2.8%) being the main degradation product as shown in Figure 3(c). NMS identity was confirmed by using NMP degradation products and process impurities standard solution mix as per Figure 3(f). An impurity profile summary is shown Table 1.

Based on previous literature by Patton and Drago [10], we hypothesized that NMP oxidative degradation products were the source of the observed pH decline in which cobalt could interact with triplet state molecular oxygen in the system to generate a reactive metal-peroxo species. These reactive species may be the source of a Class IV-type radical oxidative mechanism with NMP [11, 12] due to the susceptibility of the *α*-carbon position of lactam compounds to hydrogen abstraction [10, 22–24]. To test this hypothesis, we employed LC/-UV to quantify changes in NMP levels during its exposure to cobalt under small-scale reaction conditions. Our analytical method was developed to detect and quantify the known impurities/degradation products of NMP, including NMS as identified by various authors [10, 15–17], as well as succinimide and pyrrolidin-2-one (2P) which were observed by Friesen et al. [25]. LC profiles presented in Figure 3 show that only NMS was observed under the studied experimental conditions (Figure 3(b)). The formation of NMS was observed in previous studies [15–17] and agrees with the oxidative pathway outlined by Drago [12]. This figure also shows that nonidentified (unknown) degradation products (RRT 0.92 and RRT 1.16) were formed in high levels. By replacing the oxygen gas with inert nitrogen (Figure 3(c)), the oxidative degradation reaction was significantly suppressed showing that the main degradation products (RRT 0.92) and NMS were detected at a lower concentration compared with the oxygen-assisted oxidation. The proposed chromatographic platform is a suitable stability-indicating method as all known process impurities and main degradation products are well separated.

### 3.5. Mass Spectrometry Characterisation

To characterize the unknown degradation products, mass spectrometry analysis was conducted on both samples.  Table 2 summarizes all MS data generated which includes accurate mass measurements, fragmentation, and deuterium exchange analysis.

**Figure 4:**
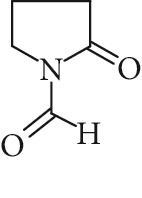
High resolution mass spectra (MS) and product ion (MS^2^) spectra of (a) compound detected as protonated mass at *m*/*z* 118.0865 Da (mass accuracy 2.2 ppm) corresponding to RRT 0.27 in the LC/UV platform with MS^2^ at *m*/*z* 88.10 and 100.11. This proposed compound could correspond to the hydrolysis of the NMP to N-methyl-4-aminobutanoic (NM4ABA) in residual water contained in NMP. (b) NMP showing parent protonated mass at *m*/*z* 100.0759 (mass accuracy of 1.02 ppm) and MS^2^ at *m*/*z* 58.07. (c) N-Methylsuccinimide (NMS) corresponding to RRT 0.63 at *m*/*z* 114.0552 Da with a mass accuracy of 2.1 ppm and main daughter ion at *m*/*z* 86.06 Da. (d) Main degradation product at RRT 0.92 (LC/UV) showed a protonated parent mass at *m*/*z* at 116.0708 Da and a product ion at *m*/*z* 73.11 proposed as 5-hydroxy-N-methylpyrrolidone (5-HNMP) with a mass accuracy of 1.5 ppm.

**Figure 5:**
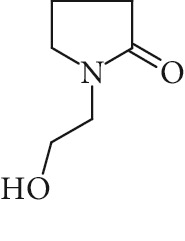
High resolution mass spectra (MS) and product ion (MS^2^) spectra of (a) RRT 1.16 (LC/UV platform) observed at *m*/*z* 114.0553 Da proposed as 1-formyl-2-pyrrolidone (FP) (mass accuracy of 2.7) with a one dominant daughter ion, at *m*/*z* 98.11 Da. (b) RRT 1.82 (LC/UV platform) was proposed as N-ethylpyrrolidone (NEP) (mass accuracy 1.8 ppm) a well-known process impurity. Mass spectrum showed a protonated mass at *m*/*z* 114.0915 Da with a MS^2^ spectrum at *m*/*z* 100.08 corresponding to a loss of CH_3_. (c) RRT 1.52 was proposed as (1-acetyl-2-pyrrolidone, 2-NEP) at *m*/*z* 130.0867 Da with a mass accuracy of 3.7 ppm and a main daughter ion at *m*/*z* 112.05 corresponding to a neutral loss of carbon monoxide (CO). (d) RRT 1.33 (split peak) was proposed as 1-(2-hydroxyethyl)-2-pyrrolidone (2-AP). Mass spectrum displayed protonated mass at *m*/*z* 128.0710 Da and main fragment at *m*/*z* 100.09 corresponding to a water loss.

**Figure 6:**
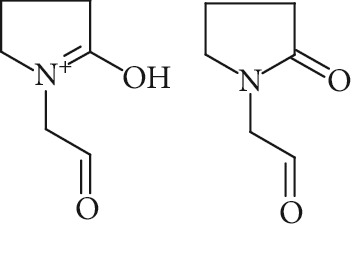
Proposed degradation chemistry mechanisms. (a) The reaction may proceed via three different pathways. Pathway I: The attack of OH radicals proceeds via a hydrogen abstraction on the CH_2_ group adjacent to the amine group of NMP being NMS the main degradant. Pathway II: The attack of OH radicals proceeds via a hydrogen abstraction on the methyl group of NMP leading to the formation of FP. Pathway III: This pathway is more speculative than pathways I and II, and it may proceed via a ring opening mechanism, leading to the formation of NM4ABA. (b) Degradation pathways for minor impurities have also been proposed and it was like the proposed pathway II. N-Methyl-4-aminobutanoic (NM4ABA), N-methylsuccinimide (NMS), 5-hydroxy-N-methylpyrrolidone (5-HNMP), 1-formyl-2-pyrrolidone (FP), 1-acetyl-2-pyrrolidone (2-NEP), and 1-(2-Hydroxyethyl)-2-pyrrolidone (2-AP).

Potential structures were proposed based on the data generated (Figures 4 and 5) and a chemical degradation pathway was suggested as shown in Figure 6. To identify as many reaction products as possible, both ESI-MS and MS/MS experiments were conducted in positive ion modes with a scan range of 50–250 Da. NMP was detected in positive ion mode at *m*/*z* 100.0759 Da with a mass accuracy of 1.0 ppm (Figure 4(b)). MS^2^ of the parent at 100.07 Da showed a main daughter ion at *m*/*z* 58 Da and a minor product ion at *m*/*z* 72 Da (Figure 4(b)). One of the main degradation products identified in LC-UV was N-methylsuccinimide (NMS) (RRT 0.63) (Figure 3(a)), this was confirmed by LC/MS in positive ion mode at *m*/*z* = 114.0552 Da with a mass accuracy of 2.1 ppm (Figure 4(c)), main daughter ion at *m*/*z* = 86 Da, and no exchangeable protons were observed (Figure 4(c)). The identification was also confirmed using a standard. The main degradation product at RRT 0.92 showed a parent ion at *m*/*z* 116.0708 Da and a product ion at *m*/*z* 73 Da (Figure 4(d)). In a review, Von Sonntag and Schuchmann [26] have shown that concomitant formation of hydroxycarbonyl and dicarboxylic compounds can occur during the oxidation of organic compounds in the aqueous phase. Moreover, Friesen et al. [25] also observed the simultaneous formation of NMS (dicarbonyl) and 5-hydroxy-N-methylpyrrolidone (5-HNMP). Therefore, the formation of 5-HNMP was proposed for the unknown degradant at RRT 0.92 based on mass accuracy (1.46 ppm), good agreement with fragmentation patterns (loss of CH_2_ CO), and deuterium exchange analysis data as one exchangeable proton was observed. The identification was also confirmed using a standard.

Regarding RRT 1.16, the same monoisotopic protonated mass as NMS was observed (*m*/*z* 114.0553 Da) (Figure 5(a)). Atkinson [27] has shown that 1-formyl-2-pyrrolidone (FP) is an important primary reaction product of NMP oxidation in the gas-phase. The monoisotopic molecular weight of FP is 113.0471 Da (mass accuracy 2.7 ppm), equal to that of NMS. MS^2^ analysis revealed the formation of one dominant daughter ion at *m*/*z* 98 Da. FP is not commercially available and could not be confirmed using a standard. The major fragmentation pathway of FP involves the loss of water. Such a fragmentation leads to the formation of a major daughter ion at *m*/*z* = 98 Da, thus confirming the “tentative” identity of FP. It is therefore likely that FP may be the unidentified primary reaction product detected in LC-UV at RRT 1.16.

The formation of a compound detected at *m*/*z* 118.0865 Da (RRT 0.27) could correspond to the hydrolysis of NMP to N-methyl-4-aminobutanoic acid (NM4ABA) in residual water contained within NMP (Figure 4(a)). The presence of the proposed molecule was suggested by Zegota et al. [28] and confirmed by further studies [29]. Finally, Drago and Riley [30] have characterized NM4ABA during the oxidation of N-alkylamides in the aqueous phase. Unfortunately, commercial standards of NM4ABA are not available; therefore, the identification was solely based on the MS data. Two deuterium exchangeable protons were observed along with daughter ions at *m*/*z* = 100 and 88 Da, respectively, and a mass accuracy of 2.2 ppm. Hence, it is likely that the peak at RRT 0.27 Da corresponds to NM4ABA. Other minor impurities observed at RRT 1.33, RRT 1.52, and RRT 1.82 have shown an oxidative degradation pathway and were not observed in a nitrogen atmosphere. As identification of minor impurities is not the main aim of this study, standard confirmation was not performed. RRT 1.82 was proposed as N-ethylpyrrolidone (NEP), a well-known process impurity. Protonated monoisotopic (*m*/*z* 114.0915 Da) mass agreed with the proposed structure with a mass accuracy of 1.8 ppm (Figure 5(b)). MS^2^ has shown a main fragment at *m*/*z* 100 corresponding to a loss of CH_3_. Deuterium exchange experiments further confirmed the proposed structure as no exchangeable protons were observed. The proposed NEP followed an oxidative degradation pathway by forming RRT 1.33 (split peak) 1-(2-Hydroxyethyl)-2-pyrrolidone (2-AP) at *m*/*z* 128.0710 Da (Figure 5(d)) and RRT 1.52 (1-acetyl-2-pyrrolidone, 2-NEP) at *m*/*z* 130.0867 Da (Figure 5(c)). Both degradations have shown good mass accuracy against suggested structures (3.02 and 3.73 ppm, respectively). Fragmentation showed a loss of CO (28 Da) for RRT 1.33 and loss of water (18 Da) for RRT 1.52, and exchangeable protons further confirmed the proposed structures.

### 3.6. Proposed Degradation Chemistry Mechanisms

Based on the identified reaction products, we suggest a mechanism of oxidation/hydrolysis of NMP in the nonaqueous phase, under oxygen conditions and cobalt-sodium Y-zeolite catalyst (Figure 6(a)) as the main degradation pathway. This oxidation is partially quenched by using an inert atmosphere. The reaction can proceed via three different pathways (Figure 6(a)). Pathway I: The attack of OH^.^ radicals proceeds via a hydrogen abstraction on the CH_2_ group adjacent to the amine group of NMP [17]. This leads to the formation of an alkyl radical which reacts with dissolved oxygen to form a peroxyradical. By analogy with the aqueous phase behavior of other peroxy radicals [25, 27], this radical can self-react to form a tetroxide, which rapidly decomposes into NMS. Pathway II: The attack of OH^.^ radicals proceeds via a hydrogen abstraction on the methyl group of NMP. This pathway leads to the formation of another alkyl radical, which then reacts with dissolved oxygen to form a peroxy radical. This peroxy radical can self-react to form a tetroxide which rapidly decomposes, leading to the formation of FP. The LC-UV analysis confirmed the importance of this pathway as FP accounted for 11.4% of NMP degradation. This second pathway was previously mentioned by Friesen et al. [25] only briefly, and to the best of knowledge, our study is the first experimental evidence of this pathway. Pathway III: This pathway is more speculative than pathways I and II. An analogous pathway was previously identified by Horikoshi et al. [31–33], who performed OH-oxidation of 2P in the aqueous phase in the presence of solid phase TiO_2_. In our conditions, i.e., in the absence of particles, this pathway did not occur. However, this pathway should be considered under real tropospheric conditions as solid phase particles of various origins are present in aqueous droplets. It proceeds via a ring opening mechanism, leading to the formation of NM4ABA. We have also proposed a degradation pathway for minor impurities (Figure 6(b)). Degradation chemistry in this case was similar to that proposed for pathway II.

## 4. Conclusion

This study has confirmed our hypothesis that NMP oxidation is present in the photolithography lift-off process under current photoresist removal operating conditions. For the first time, the pH of NMP has been monitored throughout the NMP oxidative degradation pathway on both production- and pilot-scale processes. It has been demonstrated that oxidation was shown to correlate with decreasing pH, and that it can be quenched by using an inert nitrogen atmosphere. The acidification of the chemical environment during the lift-off stage was correlated to the dissolution of transition metal layers deposited on the surface of the wafer substrate prior to the lift-off stage. We have identified pH as a key process input variable (KPIV) in photolithography, and suggest that strict processes to measure and control the pH of NMP should be implemented to avoid undesirable corrosion defects within the wafer build. The use of this novel multiplatform analytical approach will be of great benefit to process engineers in assessing problematic areas of the photolithography lift-off stage. Consequently, this study has implications for the design of microelectronic fabrication processes, the optimization of critical stages within the wafer build, and demonstrates the need for more control regarding quality, stability, and sustainability of industrially accepted materials used in the photolithography process.

## Figures and Tables

**Table 1:** Impurity profile by LC/UV.

Sample	∼RT^*∗*^ (min)	∼RRT^*∗*^	Area %
Nitrogen filled	2.27 (NMS)	0.67	2.8
	3.11	0.92	4.3
	4.49	1.33	0.6

Oxygen filled	0.91	0.27	1.1
	2.28 (NMS)	0.67	15.0
	3.10	0.92	10.5
	3.92	1.16	11.4
	5.14 (split peak)	1.52	2.1
	6.15	1.82	1.5

RT: retention time, RRT: relative retention time to NMP, NMS: N-methylsuccinimide.

**Table 2:** Mass spectrometry data summary.

Peak (RRT)	Monoisotopic [M + H]^+^	Mass accuracy (ppm)	D_2_O Ex protons	Fragments (*m*/*z*)	Proposed structures
0.27 (NM4ABA)	118.0865	2.2	2	100/88	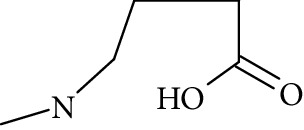
0.67 (NMS)	114.0552	2.1	0	86	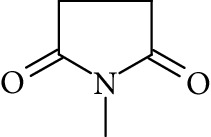
0.92 (5-HNMP)	116.0708	1.5	1	73	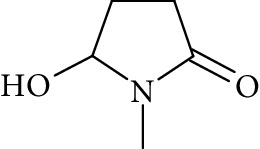
1.16 (FP)	114.0553	7.0 2.7	1	98	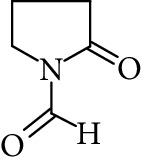
1.33 (2-NEP)	130.0867	3.7	1	112	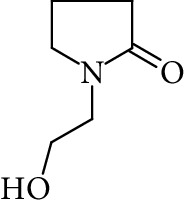
1.52 (2-AP)	128.0710	3.0	1 (first) 0 (second)	100	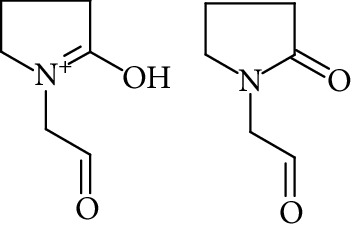
1.82 (NEP)	114.0915	1.8	0	100	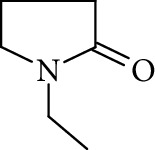
NMP	100.0758	1.0	0	58	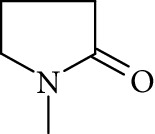

NM4ABA: N-methyl-4-aminobutanoic, NMS: N-methylsuccinimide, 5-HNMP: 5-hydroxy-N-methylpyrrolidone, FP: 1-formyl-2-pyrrolidone, 2-AP: 1-(2-hydroxyethyl)-2-pyrrolidone, 2-NEP: 1-acetyl-2-pyrrolidone, NEP: N-ethylpyrrolidone, and NMP: N-methylpyrrolidone.

## Data Availability

The raw LC/UV, ICP-OES, and LC/MS data used to support the findings of this study are available from the corresponding author upon request.

## Abbreviations

LC/MS: Liquid chromatography/mass spectrometry
LC/UV: Liquid chromatography/ultraviolet detection
NMP: N-Methyl pyrrolidone
ESI: Electrospray ionization
MS: Mass spectrometry
ICP-OES: Inductively coupled plasma optical emission spectrometry.

## Supplementary Materials

Supplementary Materials Figure S1: focused ion beam (FIB) analysis of the contact reader stack submerged in NMP after 400 s, 980 s, and 1800 s using a FEI FIB200TEM. Figure S2: experimental design used for catalytic oxidation of NMP in oxygen. Adapted from Victor et al., 2015. Table S1: ICP-OES calibration standard solution compositions. Table S2: LC/UV and LC/MS gradient profile.

## References

[1] Campbell S. A. (2001). *The Science and Engineering of Microelectronic Fabrication*.

[2] Dean A., Fitzsimmons J. A., Havas J., McCormic B., Shah P. R. (1992). Photoresist stripper.

[3] Cowie J. M. G. (1994). Wet development of polymer resists: a guide to solvent selection. *Advanced Materials for Optics and Electronics*.

[4] Moreau W. M. (1988). *Semiconductor Lithograpghy: Principles Practices and Materials*.

[5] Campbell S. A., Campbell S. A. (2001). Optical lithography. *The Science and Engineering of Microelectronic Fabrication*.

[6] Tewksbury S. K. (1989). Fabrication defects. *Wafer-Level Integrated Systems. The Kluwer International Series in Engineering and Computer Science (VLSI, Computer Architecture and Digital Signal Processing)*.

[7] Nonogaki S., Ueno T., Ito T., Nonogaki S., Ueno T., Ito T. (1998). Chemistry of photoresist materials. *Microlithography Fundamentals in Semiconductor Devices and Fabrication Technology*.

[8] Jaegar R. C., Jaegar R. C., Neudeck G. W., Pierret R. F. (1993). Lithography. *Introduction to Microelectronic Fabrication*.

[9] Jouyban A., Fakhree M. A. A., Shayanfar A. (2010). Review of pharmaceutical applications of N-methyl-2-pyrrolidone. *Journal of Pharmacy & Pharmaceutical Sciences*.

[10] Patton D. E., Drago R. S. (1993). Regenerable N-alkylamide hydroperoxide for catalytic substrate oxidation. *Journal of the Chemical Society, Perkin Transactions 1*.

[11] Drago R. S., Beer R. H. (1992). A classification scheme for homogeneous metal catalyzed oxidations by O_2_. *Inorganica Chimica Acta*.

[12] Drago R. S. (1992). Homogeneous metal-catalyzed oxidations by O_2_. *Coordination Chemistry Reviews*.

[13] Saussine L., Brazi E., Robine A., Mimoun H., Fischer J., Weiss R. (1985). Cobalt(III) alkylperoxy complexes. Synthesis, X-ray structure, and role in the catalytic decomposition of alkyl hydroperoxides and in the hydroxylation of hydrocarbons. *Journal of the American Chemical Society*.

[14] Reist H., George D. (2004). Accelerator magnet plugging by metal oxides.

[15] Gambino J., Robbins J., Rutkowski T. (2008). *Etching of Copper in Deionized Water Rinse*.

[16] Campbell H. L., Striebig B. A. (1999). Evaluation of N-methylpyrrolidone and its oxidative products toxicity utilizing the microtox assay. *Environmental Science & Technology*.

[17] Valverde N. (1988). Factors determining the rate of dissolution of metal oxides in acidic aqueous solutions. *Berichte der Bunsengesellschaft für physikalische Chemie*.

[18] Valverde N., Wagner C. (1976). Considerations on the kinetics and the mechanism of the dissolution of metal oxides in acidic solutions. *Berichte der Bunsengesellschaft für physikalische Chemie*.

[19] Masui M., Hara S., Ozaki S. (1986). Anodic oxidation of amides and lactams using N-hydroxyphthalimide as a mediator. *Chemical & Pharmaceutical Bulletin*.

[20] Hubli R. C., Mittra J., Suri A. K. (1997). Reduction-dissolution of cobalt oxide in acid media: a kinetic study. *Hydrometallurgy*.

[21] Yakusheva E. A., Gorichev I. G., Atanasyan T. K., Lainer Y. A. (2010). Kinetics of dissolution of cobalt oxides in acidic media. *Russian Metallurgy (Metally)*.

[22] Yakusheva E. A., Gorichev I. G., Atanasyan T. K., Lainer Y. A. (2012). Kinetics of the dissolution of cobalt, nickel, and iron oxides in sulfuric acid. *Russian Metallurgy (Metally)*.

[23] Gramain J.-C., Remuson R., Troin Y. (1976). A new photo-oxidation: the transformation of lactams into imides. *Journal of the Chemical Society, Chemical Communications*.

[24] Okita M., Wakamatsu T., Ban Y. (1979). Anodic oxidation of N-alkyl-lactams. *Journal of the Chemical Society, Chemical Communications*.

[25] Friesen D. A., Headley J. V., Langford C. H. (1999). The photooxidative degradation of N-methylpyrrolidinone in the presence of Cs3PW12O40 and TiO2 colloid photocatalysts. *Environmental Science & Technology*.

[26] von Sonntag C., Schuchmann H.-P. (1997). *Peroxyl Radicals in Aqueous Solution*.

[27] Atkinson, Report to ARCO Chemical Corporation, 1996

[28] Zegota H., Schuchmann M. N., Schulz D., Sonntag C. v., Radicals A. (1986). Acetonylperoxyl radicals, CH3COCH2O2: a study on the y-radiolysis and pulse radiolysis of acetone in oxygenated aqueous solutions. *Zeitschrift für Naturforschung B*.

[29] Poulain L. (2005). Micromechanical analysis of the surface erosion of a cohesive soil by means of a coupled lbm-dem mode.

[30] Drago R. S., Riley R. (1990). Oxidation of N-alkyl amides to novel hydroperoxides by dioxygen. *Journal of the American Chemical Society*.

[31] Horikoshi S., Hidaka H., Serpone N. (2001). Photocatalyzed degradation of polymers in aqueous semiconductor suspensions. *Journal of Photochemistry and Photobiology A: Chemistry*.

[32] Raghavan S., Small H., Lowalekar V. (2007). *Handbook for Cleaning/Decontamination of Surfaces*.

[33] Carnerup M. A., Åkesson B., Jönsson B. A. G. (2001). Determination of 5-hydroxy-N-methyl-2-pyrrolidone and 2-hydroxy-N-methylsuccinimide in human plasma and urine using liquid chromatography-electrospray tandem mass spectrometry. *Journal of Chromatography B: Biomedical Sciences and Applications*.

