# Supplementary material for: Assessing the Oxidative Degradation of N-Methylpyrrolidone (NMP) in Microelectronic Fabrication Processes by Using a Multiplatform Analytical Approach
Source: J Anal Methods Chem. 2020 Mar 4;2020:8265054. doi: 10.1155/2020/8265054 (PMC7073504; doi:10.1155/2020/8265054)
Supplement: Supplementary Materials — Figure S1: focused ion beam (FIB) analysis of the contact reader stack submerged in NMP after 400 s, 980 s, and 1800 s using a FEI FIB200TEM. Figure S2: experimental design used for catalytic oxidation of NMP in oxygen. Adapted from Victor et al., 2015. Table S1: ICP-OES calibration standard solution compositions. Table S2: LC/UV and LC/MS gradient profile. [file 8265054.f1.pdf]

Gavin Lennon[a], Shannon Willox[a], Ragini Ramdas[b], Scott Funston[b], Matthew Klunc, Robert Pieh[c], Stewart Fairlie[c], Sara Dobbin[d] and Diego F. Cobice[d]

[a] Queen's University Belfast, School of Chemistry and Chemical Engineering, David Keir Building, Stranmillis Road, Belfast, Antrim, BT7 1NN.

[b] Seagate Technology PLC, Springtown Industrial Estate, Londonderry BT48 0LY

[c] Seagate Technology PLC, Bloomington, Minnesota, MN 55435.

[d] Ulster University, Centre of Molecular Biosciences, Mass Spectrometry Centre, Cromore Road, Coleraine Campus, Londonderry, BT52 1SA, UK. Electronic address: d.cobice@ulster.ac.uk.

Title: Assessing the Oxidative Degradation of N-methyl Pyrrolidone (NMP) in Microelectronic Fabrication Processes by using a Multi-platform Analytical Approach

Table of content

1. **Supplementary Data**

1.1 **Supplementary figure** S2

1.2 **Supplementary tables** S4

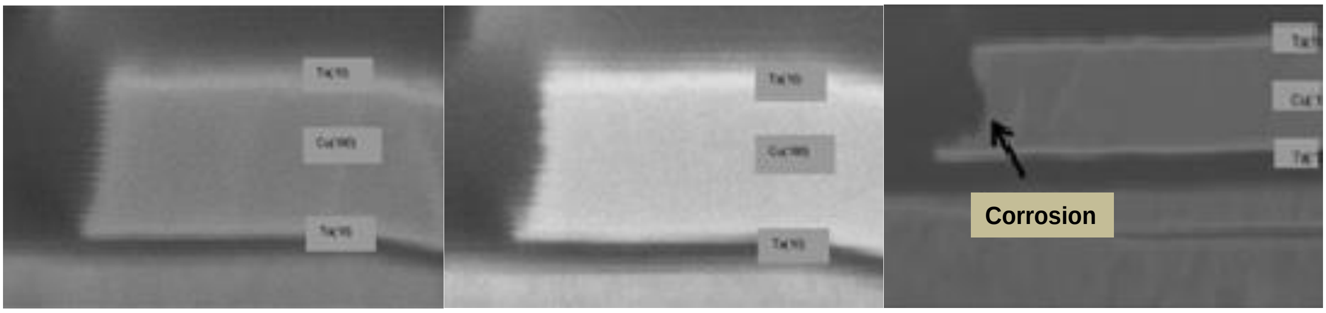

Figure S1: Focused ion beam (FIB) analysis of the contact reader stack submerged in NMP after 400 s, 980 s, and 1800 s using a FEI FIB200TEM

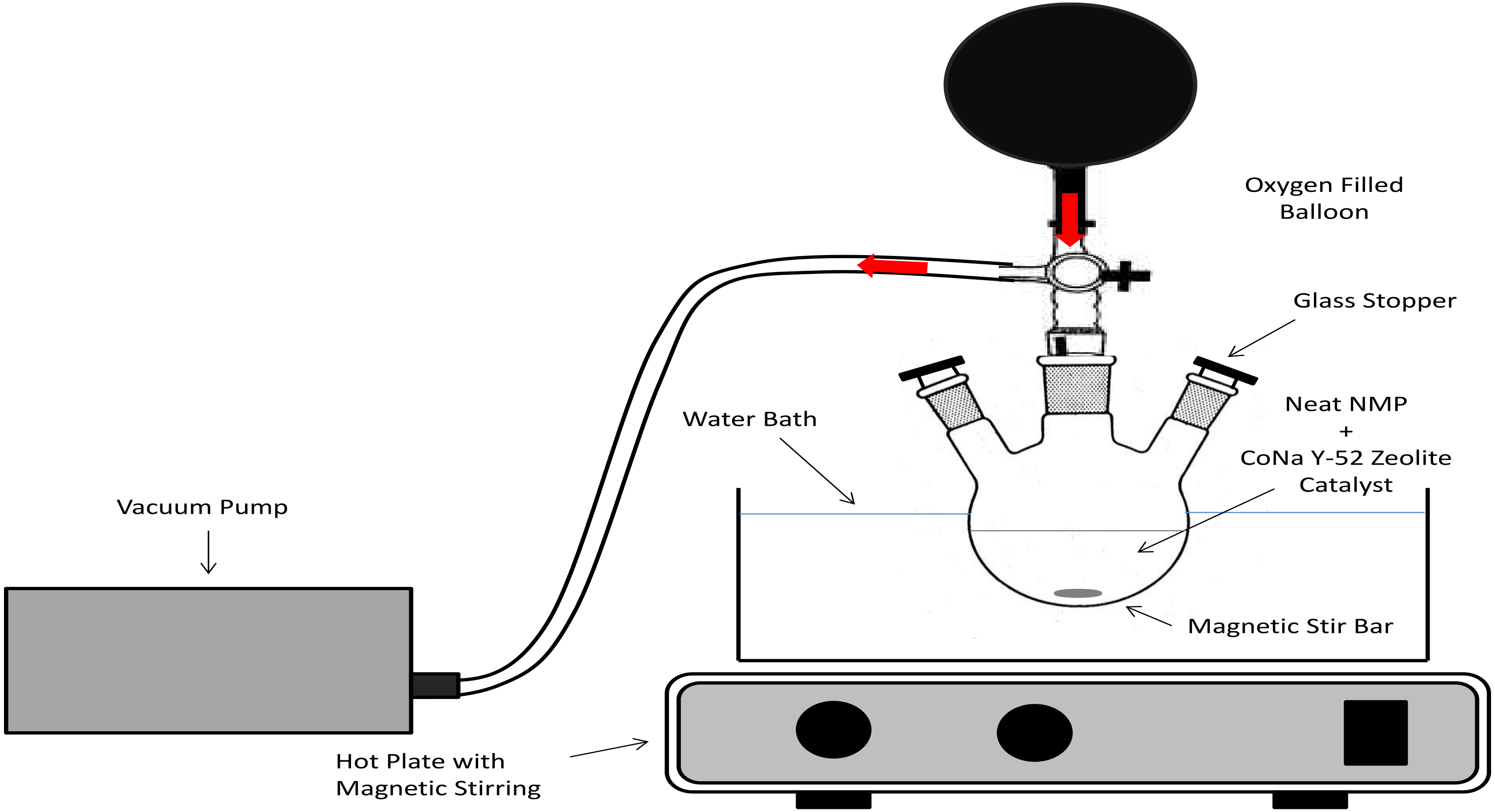

Figure S2: Experimental design used for catalytic oxidation of NMP in Oxygen. Adapted from Victor *et., 2015*.

Table S1: ICP-OES Calibration Standard Solution Compositions

Calibration Standard	Volume of NMP (mL)	Volume of Working Standard A (mL)[a]	Volume of Working Standard B (mL)[b]	Volume of Deionised Water
5	10	20	-	70
4	10	10	-	80
3	10	1	-	89
2	10	-	10	80
1	10	-	1	89

[a] High Concentration Working Standard (10 mg/L)
[b] Low Concentration Working Standard (0.1 mg/L)

Table S2: LC/UV and LC/MS gradient profile.

Minutes	Flow mL/min	Mobile Phase	
		% A	%B
0.00	0.5	98	2
0.20	0.5	98	2
0.50	0.7	98	2
5.00	0.7	70	30
9.00	1.0	60	40
9.01	1.0	98	2
